# Lossless and Near-Lossless Compression Algorithms for Remotely Sensed Hyperspectral Images

**DOI:** 10.3390/e26040316

**Published:** 2024-04-05

**Authors:** Amal Altamimi, Belgacem Ben Youssef

**Affiliations:** 1 Department of Computer Engineering, College of Computer and Information Sciences, King Saud University, P.O. Box 51178, Riyadh 11543, Saudi Arabia; aaltamimi@kacst.gov.sa; 2 Space Technologies Institute, King Abdulaziz City for Science and Technology, P.O. Box 8612, Riyadh 12354, Saudi Arabia

**Keywords:** hyperspectral images, image compression, lossless compression, near-lossless compression, remote sensing, seed generation, square rooting

## Abstract

Rapid and continuous advancements in remote sensing technology have resulted in finer resolutions and higher acquisition rates of hyperspectral images (HSIs). These developments have triggered a need for new processing techniques brought about by the confined power and constrained hardware resources aboard satellites. This article proposes two novel lossless and near-lossless compression methods, employing our recent seed generation and quadrature-based square rooting algorithms, respectively. The main advantage of the former method lies in its acceptable complexity utilizing simple arithmetic operations, making it suitable for real-time onboard compression. In addition, this near-lossless compressor could be incorporated for hard-to-compress images offering a stabilized reduction at nearly 40% with a maximum relative error of 0.33 and a maximum absolute error of 30. Our results also show that a lossless compression performance, in terms of compression ratio, of up to 2.6 is achieved when testing with hyperspectral images from the Corpus dataset. Further, an improvement in the compression rate over the state-of-the-art $k^{2}$-raster technique is realized for most of these HSIs by all four variations of our proposed lossless compression method. In particular, a data reduction enhancement of up to 29.89% is realized when comparing their respective geometric mean values.

## 1. Introduction

The wealth of information in hyperspectral images (HSIs) and increases in sensor performance have opened perspectives for a variety of applications, including space exploration, remote sensing, medical imaging, environmental monitoring, industrial quality control, and forensic science, among many others [1,2]. While the full potential of HSI techniques has not been fully explored, there is an increasing demand for this technology in the marketplace and in various aspects of life. According to Business Communications Company (BCC) research [3], the growth of the global market for HSI has increased at a compound annual growth rate (CAGR) of 15.1% for the period from 2018 to 2023.

A major problem with hyperspectral images is their immense size, collected by hundreds of contiguous spectral bands, where the size of each HSI can easily reach hundreds of megabytes. This would engender logistical problems in terms of storage, transmission, and processing. According to the Consultative Committee for Space Data Systems (CCSDS), the large data volume associated with hyperspectral images poses significant challenges for the myriad resources utilized for data processing in both onboard satellites and on-ground stations [4]. As a result, the use of efficient compression methods to decrease the size of these images without compromising their valuable information becomes mandatory. The availability of such compression techniques for hyperspectral images is a key enabler for unlocking the full potential of this powerful technology. It would pave the way for exploiting its full capability and enhancing our understanding of the world around us in meaningful ways. This motivated us further to undertake this research.

Image compression methods are mainly categorized into lossless and lossy compression techniques. Lossless compression allows for the exact reconstruction of the image at the expense of realizing modest compression ratios when compared to lossy compression methods. This is due to the theoretical boundary imposed by entropy on the lossless compression [5]. Entropy depends on the statistical nature of the source data and is defined as the average Shannon information content. Let X be the set of all possible outcomes $x_{i}$ of these source data. The entropy of the set X, denoted by H(X), is formulated as follows:

(1) $$H\left(X\right)=-\sum_{i=0}^{M-1}p\left(x_{i}\right)\cdot \log_{2}p\left(x_{i}\right),$$

where M is the cardinality of the set X; that is, M=|X|, and $p(x_{i})$ corresponds to the probability of the outcome $x_{i}$ [6]. In this work, the unit of the calculated entropy is bits, though the calculated entropy can be expressed in various units depending on the application. Accordingly, the range of H(X) is bound within [0, $\log_{2}\left|X\right|$].

Several studies have expanded on lossless and lossy compression algorithms [2,7,8,9]. Lossless compression algorithms are mainly implemented by means of prediction-based techniques, such as fast lossless (FL) [10], differential pulse code modulation (DPCM) [11], recursive least squares (RLS) [12], and the standard developed by the Consultative Committee for Space Data Systems (CCSDS), famously known as CCSDS-123 [13,14]. Predictive techniques are also employed in lossy compression algorithms, such as low-complexity predictive lossy compression (LCPLC) [15].

On the other hand, the implementation of lossy compression techniques relies heavily on employing transform-based methods such as principal component analysis (PCA) [16,17], discrete cosine transform (DCT) [18,19], discrete wavelet transform (DWT) [20], pairwise orthogonal transform (POT) [21], and the lossy compression algorithm for hyperspectral images (HyperLCA) [22]. The integer version of the transform-based techniques is also applied for lossless compression with limited results in terms of compression ratios. In this category, the integer version of the Karhunen–Loéve transform (KLT) is utilized to achieve lossless compression [23]. Similarly, the integer-based DWT is adopted in lossless compression as a part of the JPEG 2000 compression algorithm [24].

A third category, called near-lossless, achieves higher compression ratios than lossless compression while limiting the pixel distortion to a pre-defined absolute or relative error [25,26,27,28,29]. A more uncompromising definition of near-lossless limits the maximum error to the intrinsic noise of the original data produced by the used instrument or other sources, such as atmospheric correction [30]. Generally, near-lossless compression is achieved by one of the following means: (1) losslessly coding the quantized prediction error; (2) applying pre-quantization of the original image followed by lossless coding; or (3) a two-stage near-lossless coding.

The first category is the most popular due to its low complexity. Here, the typical prediction technique is the context-based, adaptive, lossless image codec (CALIC) method [31]. It can be extended to 3D-CALIC [32] and M-CALIC [33] by exploiting the correlation presented in the hyperspectral data. However, CALIC-based algorithms are not hardware-friendly, as reported by Zheng et al. [25]. Nonetheless, two compression techniques tailored to hardware implementations, promulgated by the Consultative Committee for Space Data Systems, include the near-lossless version of the algorithm (NL-CCSDS-123) [34] and the CCSDS-123-AC. The latter employs a context-based arithmetic coder (AC) and offers lower computational complexity [35]. Both of these methods use CCSDS-123 as a predictor, except that the former employs a range encoder, and the latter employs a context-based arithmetic encoder. The former encoder is a simplified version of the latter, though it yields suboptimal results [34,35].

In the second category, lossless compression is performed on a pre-quantized image. Such techniques yield poor compression performance with increasing tolerance values [27]. To bind the tolerance to a certain δ value, the quantizer step size is set to 2δ+1 [33]. The technique proposed in [36] falls under this category. It extends the existing CCSDS algorithm for Image Data Compression (CCSDS-IDC) with a pre-quantizer to increase the compression rate. The quantization in this study is carried out by employing a quantization table instead of a scaler-based quantizer.

The third category combines both lossy and lossless compression. The residual image resulting from the difference between the original image and the lossy compressed image is quantized and then losslessly encoded. A two-stage coder is proposed by Beerten et al., with the JPEG 2000 being the lossy layer [27]. The residual image is then encoded bit-plane by bit-plane using binary arithmetic coding. This allows the encoder to terminate the coding process at any arbitrary bit rate. Other studies propose pairing the lossless compression technique CALIC with a wavelet-based approach [26,37] or with JPEG 2000 [38] as the lossy layer.

In our previous work reviewing hardware-accelerated compression of remotely sensed hyperspectral images, results show that lossless compression started to gain the attention of the research community as early as 2009 [9]. Then, it increased thereafter, perhaps due to the growing demand for loss-free hyperspectral images by a myriad of research and development projects for various analysis tasks. Our investigation also shows that there is a limited number of studies on the development of near-lossless compression when compared to the rich literature on lossless and lossy compression methods [9].

Overall, the work described herein makes the following main research contributions:

- A novel lossless compression technique of remotely sensed hyperspectral images is proposed by employing our recent method of seed generation based on bit manipulation techniques [39]. Four variations are employed in our experiments using the Corpus dataset of HSIs. Our performance results yield an enhancement in data reduction that reaches 29.89% when comparing the corresponding geometric mean value with that obtained by the state-of-the-art $k^{2}$-raster method [40].
- A novel near-lossless compression of HSIs is also proposed by incorporating our published quadrature-based square rooting method [39]. A data reduction that varies from 38.90% to 39.73% is realized with a maximum relative error of 0.33 and a maximum absolute error of only 30. Since hyperspectral images with high entropies are hard to losslessly compress due to their reduced correlation, this approach can be applied with a small to negligible impact on the accuracy of the decompressed data.

The rest of the paper is structured as follows: Section 2 gives a short review of some recent works related to the compression of HSIs. Then, Section 3 describes the lossless compression of remotely sensed hyperspectral images utilizing our seed generation approach. This is followed by the proposed near-lossless compression employing our quadrature-based square rooting method in Section 4. In Section 5, we present the experimental results and compression performance obtained by both proposed compression systems. Finally, our concluding remarks and future work are provided in Section 6.

## 2. Related Work

In this section, we review some recent studies pertaining to this research focus, with a particular emphasis on works published since 2018. Some of the reviewed papers span HSI compression systems that are transform based and tensor based. Others rely on a multitude of computational techniques that include machine learning, compressive sensing, and recursive least-squares.

In the realm of machine learning and, in particular, deep learning, several algorithms have been proposed [41,42,43]. In 2019, a lossy hyperspectral image compression system that combines an onboard predictive compressor and a ground-based convolutional neural network (CNN) for image reconstruction was described in [41]. Results show that the quantization of hyperspectral data followed by the lossless mode of CCSDS-123.0-B-2 outperforms the lossy mode of the standard in terms of speed. Furthermore, a comparable rate-distortion performance is achieved by incorporating the ground-based CNN. Deep learning is also employed in lossless compression as proposed in [42] by leveraging deep recurrent neural networks (RNN) to improve the prediction accuracy of the traditional DPCM approach. Another approach based on machine learning involves the application of support vector regression (SVR) [43]. First, a 3D wavelet transform is used to simultaneously capture spatial and spectral features. Then, SVR is used to predict the behavior of the 3D wavelet coefficients. Entropy encoding, including run-length encoding and arithmetic encoding, is used to achieve a high rate-distortion performance of 40.65 dB that yields a classification rate baseline of 75.8% [43]. A lossy hyperspectral image compression algorithm, leveraging autoencoders and deep learning techniques, is described in [44]. It yields significant improvements in compression ratio and a 28% enhancement in peak signal-to-noise Ratio (PSNR). The classification accuracy remains largely unaffected by the compression process, demonstrating effectiveness in preserving image quality. In 2023, a novel hyperspectral compression network via contrastive learning (HCCNet) is proposed to enhance feature distinction [45]. Contrastive learning aims to learn useful data representations by comparing similar and dissimilar pairs of examples [46,47]. Innovative modules like contrastive informative feature encoding (CIFE) and contrastive invariant feature recovery (CIFR) preserve informative attributes resulting in significant improvements. The former, CIFE, is designed to extract and organize discriminative attributes, while the latter, CIFR, is intended to recover lost attributes caused by compression. However, a semantic gap between compressed and original images remains, indicating areas for future research.

Multiple studies explored the application of transform-based methods in the compression of hyperspectral images. In a recent study by Melián et al. [48], the information extracted from subsequent frames is reused to speed up the compression performance of the lossy compressor, known as HyperLCA. Results show a 27% reduction in the floating-point operations (FLOPs) while maintaining a comparable signal-to-noise ratio (SNR). Another study focuses on the hardware aspect of the algorithm through the introduction of HW-HyperLCA [22]. The latter utilizes integer arithmetic for high compression ratios (CRs) and compression–distortion ratios (CDRs). Further, the method supports the development of different integer versions that can be implemented for specific applications. A lossy compression scheme using three-dimensional wavelet block tree coding (3D-WBTC) exploits intra and inter-sub-band correlations is disclosed in [49]. The proposed method outperforms the three-dimensional set partition embedded block coding (3D-SPECK) strategy at low bit rates and exhibits faster decoding and encoding times. The work in [50] introduces a method to enhance three-dimensional discrete cosine transform (3D-DCT) -based compression of hyperspectral images by using a luminance transform. The approach involves two steps: initially applying the luminance transform to reduce brightness and contrast differences within spectral band groups, followed by compression using 3D-DCT and entropy encoding. Compared to the traditional 3D-DCT, this method shows improved results [50]. Tzamarias et al. introduce integer-to-integer transforms within bi-orthogonal graph filter banks to enable lossless compression [51]. Such filter banks decompose hyperspectral images into different frequency sub-bands using graph-based operations, which take into account both the spatial and spectral correlations in the data. This decomposition enables efficient compression by focusing on capturing the most relevant information in the image while discarding redundant or less critical components.

Tensor-based compression methods have gained interest for effectively managing complex multi-dimensional data, especially in hyperspectral image compression. A new approach called tensor-robust CUR, for TRCUR, aimed at addressing the challenges of compressing and denoising of hyperspectral data, which frequently suffer from quality degradation due to noise is provided in [52]. While the reported method, tensor robust principal component analysis (TRPCA), is effective, it imposes significant computational demands, especially for large datasets that may exceed memory capacity limits. To overcome this constraint, TRCUR adopts a divide-and-conquer strategy by heavily downsampling the input data to create smaller subtensors. TRPCA is then applied to these subtensors to obtain low-rank solutions. Finally, the desired hyperspectral image is reconstructed by combining these low-rank solutions using tensor CUR reconstruction. Expanding upon the concept of CUR decomposition, it is a technique where a given matrix A is broken down into three matrices: C, U, and R. Matrix C contains a small subset of actual columns from A, while R consists of a small subset of actual rows from A. Matrix U is meticulously constructed to ensure that the product C×U×R closely approximates the original matrix A [53]. Another study presents a compression method based on tensor decomposition for hyperspectral images [54]. It involves obtaining a differential representation of the data between consecutive spectral bands. Then, the first band is compressed using JPEG, while the differential data are compressed using a special mathematical operation called sparse Tucker tensor decomposition. During decoding, the compressed first band and differential data are combined to reconstruct the hyperspectral image [54].

Compressed sensing (CS) has emerged as a powerful tool for hyperspectral image compression, offering efficient reconstruction from sparse measurements [55]. Compressed sensing, combined with dictionary learning, is employed for lossless compression and reconstruction of hyperspectral images in [56]. The method involves training a BlockSparse dictionary without prior knowledge about how the training data are grouped. Then, a measurement matrix is used to compress the hyperspectral data. The final step involves reconstructing the image by using the trained dictionary incorporating classification features of the hyperspectral data. CS is also employed in [57], where sparsification of hyperspectral image and reconstruction (SHSIR) is introduced. The adopted method incorporates the robust minimum volume simplex algorithm (RMVSA) to improve the accuracy of endmember extraction while the Bregman solver is employed to boost the reconstruction accuracy. Another method called context-aware compressed sensing (CACS) is presented in [58]. This method incorporates contextual information into the process of learning the dictionary and reconstructing the hyperspectral images. Further, the study in [59] employs compressed sensing in the spectral dimension by sparsely expressing this dimension as a column vector and utilizing a small dictionary. This approach avoids repeated calculations and eliminates the effects of blocking.

The recursive least squares method stands out as a commonly employed technique for hyperspectral image compression. In this regard, the bimodal conventional recursive least-squares (B-CRLS) is designed to address issues like misalignment-induced boresight effects and blurry band predictions in hyperspectral images [60]. Due to the improved band prediction, the method achieves a comparable lossless compression performance, when compared to adaptive-length and fixed-length CRLS while providing relatively lower computational times. Additionally, two parallel methods, SuperRLS and BSuperRLS, are proposed for lossless compression of hyperspectral images in [61]. These methods involve superpixel segmentation, parallel prediction using Recursive Least-squares, and encoding residuals with an arithmetic encoder. SuperRLS offers advantages such as parallelizability, competitive compression ratios, and reconstruction of pixels under selected superpixels. BSuperRLS, having a similar structure, achieves the best compression performance with lower computational times.

Finally, the works by Chow et al. describe the compression of hyperspectral images by utilizing a $k^{2}$-raster compact data structure that achieves a compression ratio similar to classical techniques, enabling direct access without decompression [40,62]. The $k^{2}$-tree structure serves as a compact representation of the adjacency matrix for a directed graph [63]. Chow et al. also suggest that the $k^{2}$-raster structure works best when combined with directly addressable codes (DACs) [40,62]. We conclude this review of recent research works on HSI compression by summarizing the key aspects of the above-mentioned studies in Table 1.

## 3. Lossless Compression

We employ our recent method for seed generation to achieve lossless compression [39]. The main advantage of this approach lies in its reasonable time complexity based on using simple arithmetic operations, potentially making it suitable for real-time compression onboard satellites. Reduction is realized by utilizing the fact that the integer part of the square root of x requires ⌈n⁄2⌉ bits, where n is the number of bits required to obtain the binary representation of x [64]. In addition, for a uniformly distributed x∈N, the distribution of the values of $\lfloor \sqrt{x}\rfloor ,$ given by f(x), is skewed to the left and is formulated as $f(x)=2\lfloor \sqrt{x}\rfloor$. This means, for example, that there are more integers mapped to the square root value of 9 (nineteen values comprising integers from 81 to 99) than integers mapped to the square root value of 2 (five values comprising integers from 4 to 8). This fact yields a reduced entropy of the integer square roots when compared to the corresponding values of x. As a result, further reduction can be realized by employing any simple entropy encoder.

To losslessly achieve this reduction, we also need to preserve the fractional part of the square root for accurate retrieval of the original value of x. In the following section, we provide details on the computation and encoding scheme of both the integral and fractional parts of the square root to achieve lossless compression.

### 3.1. Computation of the Integral Part

The initial estimation of the square root of x is given by the seed $s_{0}$. This seed can be obtained by averaging the value of the leftmost ⌈n⁄2⌉ bits of the binary representation of x, denoted as the most significant half (MSH), and the quantity $2^{\left\lfloor n⁄2\right\rfloor }$, where n is as defined previously. This is formulated as follows:

(2) $$s_{0}=0.5\times \left(MSH+2^{\left\lfloor n/2\right\rfloor }\right).$$

**Figure 1:**
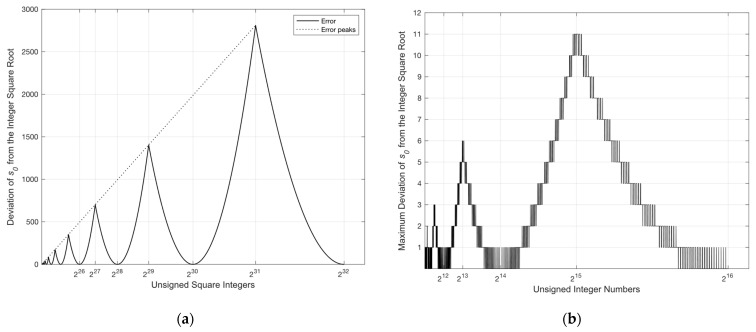
(**a**) A scaled plot showing the deviation of the generated seed from the correct square root for square numbers up to $2^{32}-1$; (**b**) a discrete plot showing the maximum deviation of $s_{0}$ from the integer square root for numbers up to $2^{16}-1$.

We observe from the above equation that the seed $s_{0}$ estimates the integer square root of an unsigned integer x using only two operations: one addition followed by a single-bit right shift. This generated seed is an accurate estimate of the integer square root of unsigned integers up to eight bits. Beyond this size, a deviation from the correct square root begins to follow a pattern of connected parabola-like curves (PLC) with a different focal point for each side of the curve (see Figure 1a). The vertices (v) of the PLCs are located at the even powers of two on the x-axis whereas the peaks, denoted by $x_{peak}$, are seen at the odd powers of two [39].

The magnitude of each one of the peaks, given by $y_{peak}$, follows a first-degree polynomial provided by the equation below:

(3) $$y_{peak}=0.0572s_{peak},$$

where $s_{peak}$ is the calculated seed of $x_{peak}$ using Equation (2). For instance, the calculated seed of $x_{peak}=2^{23}$ is equal to 3072 with a deviation of +175.7 from the correct square root value of 2896.3. This error can be estimated using Equation (3), producing the same value of 175.7=0.0572×3072. It follows that by knowing both $x_{peak}$ and $y_{peak}$, the focal point (f) for each side of the PLC is calculated as follows:

(4) $$f={\left(x_{peak}-v\right)}^{2}/\left(4\times y_{peak}\right).$$

Thus, by incorporating the equation of the vertical parabola, the deviation of the estimated square root (denoted by err) of an unsigned integer x is obtained as next:

(5) $$err={\left(x-v\right)}^{2}/\left(4\times f\right).$$

Since the bit depth of the selected HSIs does not exceed 16 bits, we consider two approaches to handling this error [65]. We can either compensate for the error over the entire bit depth or completely avoid it by processing the acquired data as two distinct bytes.

#### 3.1.1. Error Compensation

As noted from Figure 1, the generated error is always positive. This means that the estimated integer square root is always equal to or greater than the integer square root of $x\;(s_{0}\geq \lfloor \sqrt{x}\rfloor )$. We compensate for the generated error by utilizing the following observation. There are $2s_{i}$ non-square integers between every two consecutive square integers $s_{i}^{2}$ and $s_{i+1}^{2}$. This is derived as follows:

(6) $$\begin{array}{l} s_{i+1}^{2}-s_{i}^{2}-1={\left(s_{i}+1\right)}^{2}-s_{i}^{2}-1\\ =s_{i}^{2}+2s_{i}+1-s_{i}^{2}-1\\ =2s_{i}. \end{array}$$

**Figure 2:**
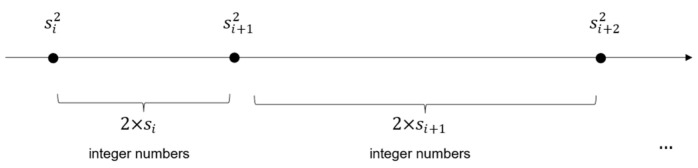
The distance between consecutive integer square numbers increases relative to their corresponding square roots.

In Figure 2, we illustrate this increasing distance between consecutive square integers relative to their corresponding square roots. According to the figure, a single step from the integer square root $s_{i}$ to $s_{i+1}$ translates to a distance of ${2s}_{i}+1$ between their corresponding square integers. Therefore, a deviation of d in the plane of integer square roots translates to a distance of $\sum_{i}^{i+d-1}\left(2s_{i}+1\right)$ in the plane of unsigned integers.

In order to find the desired square root $s_{i}$, we roll back from the deviated value $s_{i+d}$ while knowing the value of x. This is achieved by first computing the difference between $s_{i+d}^{2}$ and x. Then, from the squared value of $s_{i+d}$, we roll back a distance of $\sum_{i}^{i+d-1}\left(2s_{i}+1\right)$ towards x one leap at a time until the difference is no longer positive. For instance, the calculated seed for the square number x=8464 is 97. This value is deviated by +5 from the correct integer square root s=92. Knowing the distance between x and $97^{2}$, we can compensate for this deviation by first rolling back a distance of 2×96+1. If the distance remains positive, we subtract one from the estimated square root and repeat the process. This is performed multiple times until the correct integer square root is reached. This example is detailed below by showing the roll-back of a distance of ${945(=97}^{2}-8464)$ one leap at a time:

**Step 1:** 945−2×96+1=752,

**Step 2:** 752−2×95+1=561,

**Step 3:** 561−2×94+1=372,

**Step 4:** 372−2×93+1=185, and

**Step 5:** 185−2×92+1=0.

Thus, the correct integer square root value of 92 is reached when the remainder is equal to zero. However, the remainder zero is reached here because x is a perfect square. For non-square integers, the correct integer square root is reached when the remainder is less than zero. The example below shows the same procedure employing the non-square integer x=24576. The generated seed $s_{0}$ for x=24576 is 160. As a result, the distance between $s_{0}^{2}$ and x is $160^{2}-24576=1024$. That is, we can apply the rollback as follows:

**Step 1:** 1024−2×159+1=705,

**Step 2:** 705−2×158+1=388,

**Step 3:** 388−2×157+1=73, and

**Step 4:** 73−2×156+1=−240.

The iterative subtraction is stopped when the remainder is less than zero giving the correct integer square root value of 156. We can generalize the above by stating that the correct integer square root is reached when the remainder is less than or equal to zero. We formulate the procedure of calculating the integer square root in Algorithm 1, for n>8 bits, as given below:
**Algorithm 1.** $\mathrm{Calculation}\;\mathrm{of}\;\mathrm{the}\;\mathrm{integer}\;\mathrm{square}\;\mathrm{root}\;\mathrm{of}\;x\;\mathrm{by}\;\mathrm{rolling}\;\mathrm{back}\;\mathrm{from}\;s_{0}$.**Input:** $x,s_{0}$ **Output:** s // integer square root of x **Initialization:** $s_{i}\leftarrow s_{0}$ $D\leftarrow s_{i}^{2}-x$ **While**  (D>0) **Do** $s_{i}\leftarrow s_{i}-1$ $D\leftarrow D-(s_{i}\ll 1)-1$ **End Do** $s\leftarrow s_{i}$

To reduce the computational complexity of finding the correct integer square root, we suggest computing the distances ($2\times s_{i}+1$) in parallel. As illustrated in Figure 1b, the maximum deviation from the correct integer square root of a 16-bit unsigned integer is +11, located at $x=2^{15}$. Therefore, we suggest computing the worst-case scenario (11 distances) at once and then serializing the jump using subtraction. As per [66], the number of cycles required by the integer instructions of Intel Pentium and Pentium MMX are as follows: one clock cycle for addition or subtraction, one clock cycle for comparison, one cycle for a shift operation, and 11 cycles for multiplication. As a result, computing the total distance $s_{i}^{2}-x$ requires 12 cycles. Then, the eleven instances of $s_{i}-1$ are unrolled and calculated in parallel within one cycle. This is followed by two cycles to calculate the jumps, $\left(s_{i}\ll 1\right)+1$, simultaneously. Finally, the conditional update of the D value requires 11 cycles for subtraction and 12 cycles for comparison. In addition to the cost of two cycles incurred in calculating the seed $s_{0}$, we obtain a total of 40 clock cycles for calculating the correct integer square root for a 16-bit unsigned integer.

#### 3.1.2. Error Avoidance

The generated error can be completely avoided by processing the 16-bit values of the hyperspectral data byte by byte. This produces an accurate estimate of the integer square root directly by using Equation (2). We note here that the sparsity of the hyperspectral data is expected to increase after their partitioning into 8-bit chunks, especially for decorrelated data. We define sparsity of hyperspectral data to mean the percentage of zero elements comprised within the image [67]. Handling zeros before square rooting avoids unnecessary computations. Further, it improves the compression ratio by shortening long streams of zeros. This is achieved by removing all the zero bytes, leaving a single-bit indicator that instructs the decoder on how to interpret the forthcoming bits. A compression could be obtained only when the following condition related to sparsity is satisfied. Let p be the percentage of having all zero bytes in the partitioned data. Removing the zero elements reduces the average number of bits to (1−p)×9+p×1=9−8 p. In order to obtain any reduction in this value, it is desired to have:

(7) 9−8p<8⇒p>0.1250.

This means that the number of zero elements must occupy more than 12.50% for any reduction to be obtained using the aforementioned approach. A similar argument applies to unpartitioned hyperspectral data in the following way

(8) $$\left(1-p\right)\times 17+p\times 1<16\Rightarrow p>0.0625$$

In this case, the percentage of zero elements is halved to 6.25% of the 16-bit values for any compression to be realized. Further reduction can be obtained by employing the run-length encoder (RLE) as the occurrence of the zero elements becomes significant after data decorrelation and partitioning. To maintain longer streams of zeros, we suggest processing the odd bytes first, as they are mostly zeros after the preprocessing stage. This processing scheme yields a greatly varying length of zero streaks. Therefore, to efficiently encode these lengths, the first vector of the RLE holds the unary code that indicates the number of bits required to represent each length. The second vector holds the binary representation of these lengths. For example, the numbers: 365,698; 2; 356; 2; and 5254 represent the first five lengths of the alternating zero and non-zero streaks of the Mt. St. Helens image after preprocessing. The number of bits required to represent each length is 19, 1, 9, 1, and 13, respectively. We clarify here that the use of a single bit is sufficient to indicate two possible lengths (1 and 2) since the length value cannot be zero in this case. This also impacts the rest of the length values as their corresponding binary representations are decremented by one. Thus, the first vector contains the unary codes:

1111111111111111110, 0, 111111110, 0, 1111111111110.

And the binary vector contains the following values:

1011001010010000001, 1, 101100011, 1, 1010010000101.

Consequently, the stream of indicator bits is reduced from 371,312 bits (=365,698+2+356+2+5254) to 86 bits (=2×19+2×1+2×9+2×1+2×13) only, for this specific example.

### 3.2. Computation of the Fractional Part

To encode the fractional part, we utilize the same observation formulated in Equation (6). As presented in Figure 2, it is evident that the integer numbers $s_{i}^{2}\leq x<s_{i+1}^{2}$ share the same integer square root $s_{i}$. However, to differentiate between the fractions that correspond to each number in this range, we need $\left\lceil \log_{2}2s_{i}\right\rceil$ bits. For instance, there are eight non-square integers (2×4) between the square integers 16 and 25 ($4^{2}$ and $5^{2}$). These eight values share the same integer square root value of 4, and we can differentiate between them by using three bits to indicate eight distinct fractions. In other words, the fractional part of the square root of x can be encoded as the distance of x from the nearest square number $s_{i}^{2}$, where $s_{i}^{2}<x$. Calculating the distance costs one multiplication ($s_{i}\times s_{i}$) and one subtraction ($x-s_{i}^{2}$). Note that the distance between every two consecutive square integers decreases as these integers become smaller. Hence, more compression can be realized. This can be achieved by utilizing the correlation presented in the hyperspectral data. The number of bits required to represent the fractional part according to the value of the integer square root $s_{i}$ is depicted in Table 2.

There are cases where the number of non-square integers bound between two square integers $s_{i}^{2}$ and $s_{i+1}^{2}$ is exactly equal to $\log_{2}2s_{i}$, which leaves no room to include the zero fraction that corresponds to the perfect square root $s_{i}$. For instance, all the integers x within the set {4, 5, 6, 7, 8} map to integer square root value of 2. To differentiate between the fractions that correspond to each integer, we need more than two bits to include the zero fraction that corresponds to the integer number 4. This occurs consistently when the integer square roots are powers of two, e.g., $s_{i}=1,2,4,8,\ldots ,etc$. Here, we demonstrate another advantage of eliminating the zero bytes beforehand. To avoid adding an extra bit, perfect squares that are powers of two are encoded with four zeros in the integer part. This instructs the decoder to interpret the next fractional bits as a unary code. Therefore, the cost of the fractional part, represented by the number of bits, remains consistent within each range. That is, the number of bits allocated for the fraction is always $\left\lceil \log_{2}2s_{i}\right\rceil$ for all $s_{i}$ values. Table 3 below presents the codewords of the proposed lossless square root-based encoder. For $s_{i}$ in the form $2^{m}$, m represents the number of ones in the unary code of the fractional part. For example, when $s_{i}=4=2^{2}$, we have m=2 and the corresponding unary code of the fractional part is 110.

### 3.3. Preprocessing

A typical lossless compressor consists of a preprocessor followed by an entropy encoder [68]. The preprocessor’s main function is to decorrelate the input data, which is then passed to the entropy encoder. Decorrelation can be achieved by means of prediction [25,62,69,70,71,72] or transform-based techniques [51,73,74,75,76]. According to [69], techniques that use lookup tables and vector quantization are also categorized as prediction-based since both types are used to generate a prediction of the data. The best predictor is the one that yields the lowest entropy and is easy to implement. Predictive techniques offer low computational complexity and moderate memory requirements [25]. In general, the transform-based techniques are more successful in lossy compression. This is due to the fact that these methods must be integer-based to achieve lossless compression, which in turn may compromise their ability to decorrelate [69].

As it is well known, the use of prediction techniques may produce negative residuals. Thus, to avoid passing such residuals to the square root computation step, a simple mapping technique, reported in [70], is employed to maintain positive values. This technique maps a residual r into an unsigned integer by utilizing the formula below for the mapping M(r):

(9) $$M\left(r\right)=\left\{\begin{array}{c} 2r\;,\;\;\;\;\;\;\;\;\;\;\;\;\;r\geq 0\\ 2\left|r\right|-1\;,\;\;\;\;\;\;\;\;r<0. \end{array}\right.$$

We note here that the use of Equation (9) increases the range of the values by one bit. In particular, the maximum residual for a 16-bit hyperspectral image of ($2^{16}-1$) maps to ($2^{17}-2$) with the minimum being equal to zero. Since 17 is not a multiple of 8, partitioning it into 8-bit blocks leaves an extra bit that requires additional handling. A better solution is to employ a bitwise exclusive-or (XOR) operation to decorrelate hyperspectral data. The application of the XOR operation as a decorrelation technique for improved lossless compression is detailed by Cooper in [77]. Generally, the correlated data tend to have similar values in their most significant bits. Therefore, the use of the XOR operation sets the most significant bits into zeros, resulting in a lower data entropy while maintaining positive values within 16 bits. The goal is to XOR the adjacent bands $B_{i}$ of each line of the acquired scene, except for the first band $B_{0}$, as indicated by Equation (10) next. Then, the original data can be retrieved at the decoder by repeating the XOR operation starting from the first band. This is similar to the use of XOR operation in data encryption [78], where the original message is XORed with the key to generate the ciphered message. Afterward, the original message is recovered by XORing the ciphered message with the same shared key. In our case, the first band $B_{0}$ corresponds to our key. Hence, we have:

(10) $${\hat{B}}_{i}=B_{i}⊕B_{i-1}\;,\;\mathrm{for}\;i>0,$$

where ${\hat{B}}_{i}$ and ⊕ represent the decorrelated band and the XOR operation, respectively. Consequently, this procedure offers a layer of security for critical data, which can be achieved by ciphering the first band of each acquired line of the scene. Hyperspectral data tend to have stronger spectral than spatial correlation [79]. Nonetheless, we will investigate the impact of both spectral and spatial decorrelation by employing the bitwise XOR operation in Section 5.

### 3.4. Postprocessing

As mentioned earlier, the entropy of the integer square roots $\left\lfloor \sqrt{x}\right\rfloor$ is significantly smaller than the entropy of the x values. A simple entropy encoder can be utilized to further reduce the total number of bits. We suggest using the well-known Rice coding technique [80] to map the most frequent values into a smaller number of bits. Rice coding is preferable when the data to be compressed follow a geometric statistical distribution [68]. This distribution is usually determined after data have been processed in an earlier stage. Typically, a Rice code of the binary representation of x is obtained by concatenating the unary representation of the quotient ($q=x/2^{k}$) and the least significant k bits of x [81].

### 3.5. Lossless Encoder/Decoder

**Figure 3:**
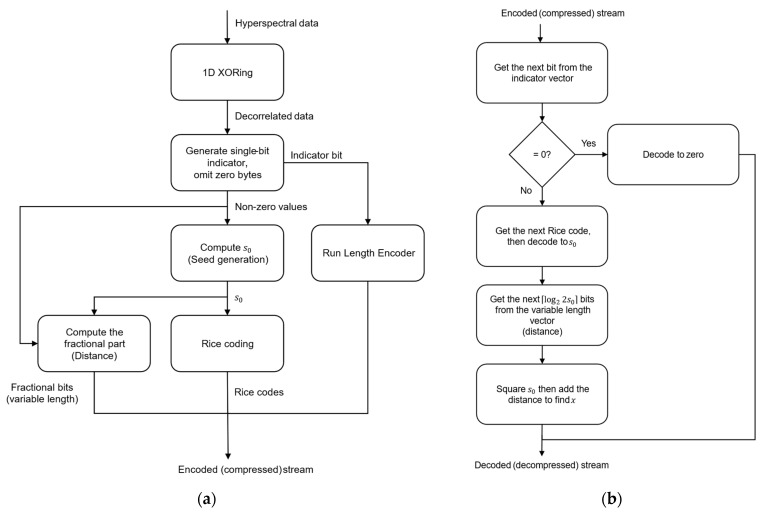
Proposed lossless compression system: (**a**) The lossless compressor contains three main stages: (1) preprocessing using one-dimensional XORing; (2) computing $s_{0}$ based on the seed generation method; and (3) postprocessing of the fixed length integral part using Rice codes. (**b**) Steps performed by the decoder of the proposed lossless compressor to reconstruct the original data stream.

The flowchart of the proposed lossless compressor is depicted in Figure 3a. The diagram highlights its three main stages: (1) preprocessing by employing one-dimensional XOR operation; (2) square rooting by means of seed generation; and (3) postprocessing by utilizing Rice codes. The decoder, presented by Figure 3b, receives the encoded data in a structure of three vectors: (1) the indicator vector (to be decoded bit by bit); (2) the Rice codes vector (to be decoded code by code); and (3) the variable length vector, where the number of bits to be processed is determined by the deciphered value of the Rice code, i.e., the value of $s_{0}$. Next, we provide in Algorithms 2 and 3 the pseudocodes for the lossless encoder and decoder, respectively.
**Algorithm 2.** Pseudocode for the compressor part of the proposed lossless compression.**Input:** x // hyperspectral data **Outputs:** vecRice, // a vector that stores the calculated seed values. vecFrac, // a vector that stores the calculated fractions. vecRLE, // a vector that holds the counts of consecutive runs of zero and nonzero values. vecUnary. // a vector that holds the variable unary codes corresponding to the number of bits of each count. **Initializations:** $x_{0}\leftarrow 0$, // the initial value to be XORed with the first element of x. ${nZ}_{0}\leftarrow 0$, // initialize the first nonzero value with 0. cnt←1, // counts the number of consecutive runs. PO2←2, // to calculate the required number of bits for each run. nVar←11, // the required number of bits for each run. n← the number of bits required to represent x. done ← 0 **For all** $x_{i}$ in x **Do** **1. Preprocessing** $xored\;\leftarrow \;x_{i}\;⊕\;x_{i-1}$ // perform exclusive-or operation. **If** xored>0 **Then** ${nZ}_{i}\leftarrow 1$ **2. Calculation of the integral part** MSH← the leftmost ⌈n/2⌉ bits of xored$$Q\leftarrow 2^{\left\lfloor n/2\right\rfloor }$$seed←(MSH+Q)≫1 riceCode←mappedRice(seed) vecRice.add(riceCode) **3. Calculation of the fractional part** $m\leftarrow \left\lceil \log_{2}\left(2\cdot seed\right)\right\rceil$ $Fr\leftarrow xored-{seed}^{2}$ // The fraction encoded as the distance between the xored value and the squared value of the seed        **If** Fr>0 **Then**        Fr ←Fr−1        **Else**        Fr ←Unary(seed)        seed ←0        **End If** ${Fr}_{out}\leftarrow$ the rightmost m bits of Fr $\mathrm{vecFr}.\mathrm{add}({Fr}_{out}$) **Else** ${nZ}_{i}\leftarrow 0$ **End If** **4. Run length encoding of** nZ **If** ${nZ}_{i}={nZ}_{i-1}$ **Then** cnt←cnt+1        **If** cnt=PO2 **Then**        PO2 ← PO2≪ 1        nVar← nVar+1        **End If** **Else** vecRLE.add(cnt) vecUnary.add(nVar) cnt ← 1 PO2 ← 2 **End If** **End Do** **If** done **Then** vecRLE.add(cnt) vecUnary.add(nVar) **End If**

**Algorithm 3.** Pseudocode for the decompressor part of the proposed lossless compression.**Inputs:** vecRice, vecFrac, vecRLE, vecUnary. **Output:** x // reconstructed hyperspectral data. **Initializations:** nVar← the number of bits derived from the next unary code in vecUnary. cnt← the run length obtained by interpreting the next nVar bits from vecRLE. Flag← 0 **For all** cnt in vecRLE **Do**        **While** cnt>0 **Do**        **If** Flag=0 **Then**        $x_{i}\;\leftarrow \;0$        **Else**        riceCode ← get the next rice code from vecRice.        seed← decode (riceCode)                **If** seed=0 **Then**                seed← the value of the next unary code from vecFrac                Fr←0                **Else**                $m\leftarrow \left\lceil \log_{2}\left(2\cdot seed\right)\right\rceil$                Fr← get the next m bits from vecFrac.                Fr ← Fr+1                **End If**$$x_{i}\leftarrow {seed}^{2}+Fr$$        **End If**        cnt←cnt−1        **End Do** Flag ←not Flag **End Do**


The sequential time complexity of the lossless compression algorithm is primarily dictated by the corresponding time complexities of the preprocessing, seed generation, fraction calculation, and post processing stages. The total time complexity can be approximated as O($n^{3}$), where n represents the number of values along each of the three dimensions of the input hyperspectral image. We assume, for the sake of simplicity, that each HSI is represented by a cube. The algorithm begins by XORing each value of the input image with its adjacent one, resulting in a complexity of O($n^{3}$) as a result of traversing the entire input data. Subsequently, the XORed values undergo seed generation, where a single shift and add operations are applied. This requires a constant complexity for each pixel value along all n bands. Thus, a total of O($n^{3}$) operations are needed to complete this step. The calculation of the corresponding fractional part also entails a cubic complexity, involving squaring the generated seed and subtracting it from the XORed value. For the postprocessing stage, the worst-case time complexity of run-length encoding is O($n^{3}$). This is because in the worst-case scenario, every element in the input data would be unique; hence requiring each element to be encoded separately. Finally, the direct mapping of Rice codes using lookup tables would exhibit a time complexity of O($n^{3}$) since it operates on each generated seed value.

## 4. Near-Lossless Compression

We aim in this part of our research to utilize our method for computing the square root value in order to achieve near-lossless compression of remotely sensed hyperspectral images [39]. Square rooting depends on exploiting the concept of quadrature. In this regard, the quadrature problem of a plane figure involves geometrically constructing a square of the same area, hence the name “quadrature” [82].

**Figure 4:**
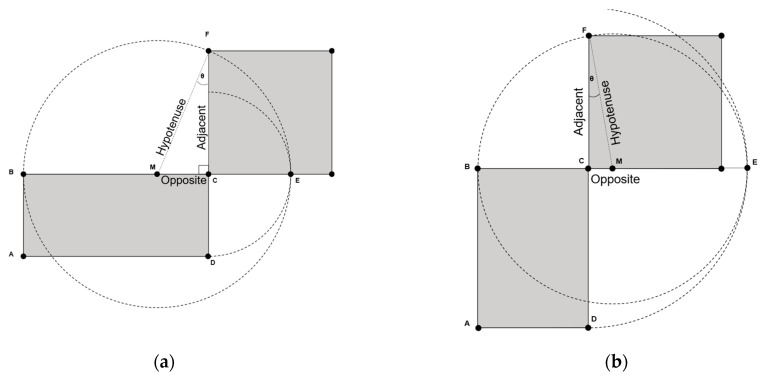
The quadrature of a plane rectangle ABCD: (**a**) The case when $s_{0}$ is the longer side of the rectangle; (**b**) The case when $s_{0}$ is the shorter side of the rectangle. In both cases, segment BC is equal to $s_{0}$.

Let x be the area of the rectangle ABCD (see Figure 4). The generated seed $s_{0}$ represents one side of the rectangle (segment BC). The other side is simply obtained by dividing x over $s_{0}$. The average of both sides of the rectangle produces the hypotenuse of the right triangle ⊿MCF. The goal is to find the length of the adjacent segment that represents the square root value. The opposite segment is calculated as the difference between $s_{0}$ and the hypotenuse. Given both the hypotenuse and the opposite segments of this right triangle, we can calculate sin⁡θ from which we can obtain the angle θ. Ultimately, the length of the adjacent segment is obtained by multiplying the hypotenuse by the cosine value of the angle θ. The pseudocode of the quadrature-based square rooting method is described next in Algorithm 4 [39].
**Algorithm 4.** The quadrature-based method to compute the square root value of x.
**Inputs:** $x,s_{0}$ **Output** : s // square root of x. **Initialization:** $BC\leftarrow s_{0}$ CD←x/BC M←0.5×(BC+CD) CM←BC−M sin⁡θ←CM/M cos⁡θ←retrieved from a lookup table utilizing sin⁡θ.  s←cos⁡θ×M

**Figure 5:**
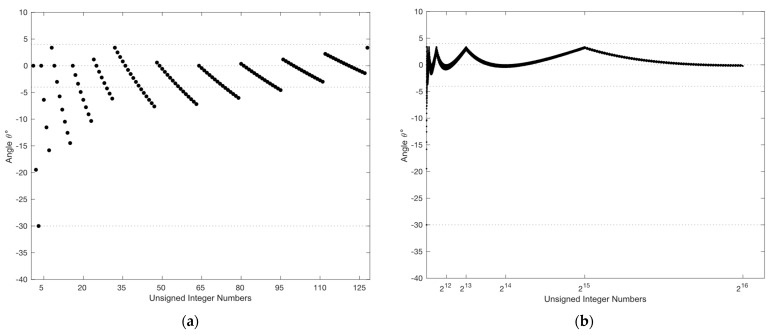
(**a**) Zoomed plot of angle θ versus x showing that the worst-case scenario occurs within the first 95 values of x, with $\theta \in \;\left[{-30}^{\circ },\;4^{\circ }\right)$; (**b**) The range of θ as a function of x for unsigned numbers up to $2^{16}-1$.

The angle θ is equal to zero when the hypotenuse and adjacent segments are coincident; i.e., both segments are of equal lengths. This means that the shape, with which we started, is a perfect square and $s_{0}$ is an accurate estimate of the square root. Less accuracy of $s_{0}$ translates to a wider deviation of θ from $0^{{}^{\circ}}$. The worst-case scenario is when the hypotenuse is almost perpendicular to the adjacent side with an angle of nearly $\pm 90^{{}^{\circ}}$. Simulation results, presented in Figure 5a, show that by employing the seed $s_{0}$, the worst-case scenario of θ is limited to ${-30}^{{}^{\circ}}$ [39]. Since both sin⁡θ and $\sin\left(-\theta \right)$ map to the same cosine value, the polarity of the angle θ has no impact on the calculated length of the adjacent segment, which corresponds to the square root value.

The value of sin⁡θ ranges between 0 and 0.5 since $\theta \in \left[{-30}^{\circ },4^{\circ }\right)$. Using a step size of 0.01 quantizes this range into 51 values. Our objective is to use these values to directly address the corresponding cosine value that is required to calculate the square root. Based on our study in [39], we found that only 24 values are actually accessed via the lookup table resulting in a corresponding utilization rate of 37.5%. These 24 variations of the index can be represented using 5 bits. However, better reduction can be achieved when using unary coding for the index part of the code. This is demonstrated by Figure 5b since most of the x values revisit the narrow range of $\theta \in \left({-4}^{{}^{\circ}},4^{{}^{\circ}}\right)$ and only a few values of x expand towards $\theta =-30^{{}^{\circ}}$. An extra reduction could be obtained by utilizing the fact that some combinations of the indices and $s_{0}$ never occur. For instance, index 6 is only reached by seven variations of $s_{0}$ and index 10 is only reached by three. Table 4 shows the possible number of variations of $s_{0}$ that can access each value of these 24 indices.

As a result, instead of encoding $s_{0}$ with fixed-length 8-bit codes, variable-length codes are used that do not exceed eight bits in the worst case. For indices ≥14, the decoder directly infers the value of the integer square root. The length of the concatenated code herein is equal to zero. Mapping $s_{0}$ values to the reduced codes is achieved using lookup tables. Since the number of bits remains at eight bits for the index value of zero, mapping in this case would only translate to an extra lookup table. Accordingly, the mapping requires a structure of lookup tables limited to only the indices in the range between 0 and 14 exclusively (0<index<14). These lookup tables are distributed as follows: (1) five lookup tables of size 256 bytes each for the range 1≤index≤5; (2) another five lookup tables of 16 bytes each for 6≤index≤10; (3) and for the range 11≤index≤13, we need only three lookup tables of eight bytes each. This results in a total of 1.4 KB for the entire structure of lookup tables. Table A1, provided in the Appendix A, shows the values of $s_{0}$ that map to each index. This is followed by Table A2, which encloses the MATLAB code used to generate Table A1. Table 5 below gives an example that illustrates the encoding of a sequence of ten 16-bit unsigned integers x.

Based on the previous table, the 16-bit binary representations of the original stream of x values are as follows:

0000000000011101, 0011000000011101, 0101100000011100, 0111100100011011, 0001010100011011, 0000011100011010, 0101001000011001, 1111001000010110, 0011101100010101, 0010100000010100.

Therefore, the corresponding codewords that employ the quadrature-based square rooting method are:

11111110000, 001001011, 100111110, 11110000110, 001000000, 11100010110, 001101101, 010010110, 001010110, 1100111000.

The unary code for the resulting indices is underlined. The binary representation that follows is the order of $s_{0}$ within a given index, as provided in Table A1. The number of bits required to represent the order is determined in advance by utilizing Table 4. For instance, the unary code of index 7, which is equal to 11111110, instructs the decoder to parse the next three bits to capture the value of $s_{0}$, which happens to be the first element for index 7, as depicted in Table A1.

### Near-Lossless Encoder/Decoder

**Figure 6:**
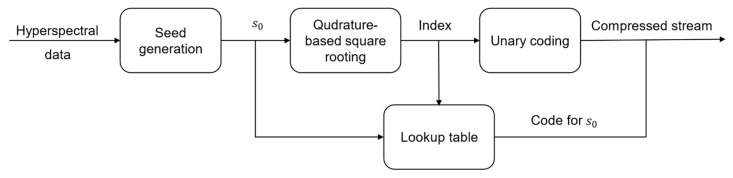
Flowchart of the proposed near-lossless compression employing our method of quadrature-based square rooting.

The flowchart of the quadrature-based encoder used to achieve near-lossless compression is exhibited in Figure 6. Starting with the hyperspectral image as an input, we apply seed generation to obtain the value of $s_{0}$. Next, the quadrature-based square rooting method is employed to compute the index value. Both the index and $s_{0}$ values are then used to obtain the encoded value of $s_{0}$ from the lookup table structure described earlier. Finally, the unary code of the index value and the code obtained from the corresponding lookup table are then concatenated to generate the compressed stream. In our case, we note that the use of decorrelation by means of either prediction or XORing may hinder the compression performance. This is due to the fact that decorrelation would reduce the majority of $s_{0}$ values, which yields smaller values of θ and thus limits the indices to the first few. Consequently, the quadrature-based encoder would not be able to benefit from the distribution presented in Table A1.

As described in Algorithm 4, the square root value is obtained by multiplying the hypotenuse by the cosine value of θ. The hypotenuse length, denoted by M, is a floating-point number, yet $s_{0}$ is an integer. Therefore, by employing the concatenation of $s_{0}$ with its corresponding index value, we would obtain better compression performance and maintain integer-based computations for the encoder. This is instead of relying on the floating-point value provided by the hypotenuse. Consequently, the last two steps of Algorithm 4 shall be assigned to the decoder on the ground station. The value of M is generated at the decoder by dividing the value of $s_{0}$ by $\left(1+\sin\theta \right)$. This is derived as follows:

(11) $$\begin{array}{l} \sin\theta =\frac{CM}{M}\;\mathrm{and}\;CM=s_{0}-M\\ ⟹\sin\theta =\frac{s_{0}-M}{M}=\frac{s_{0}}{M}-1\\ ⟹M=s_{0}/\left(1+\sin\theta \right). \end{array}$$

**Figure 7:**
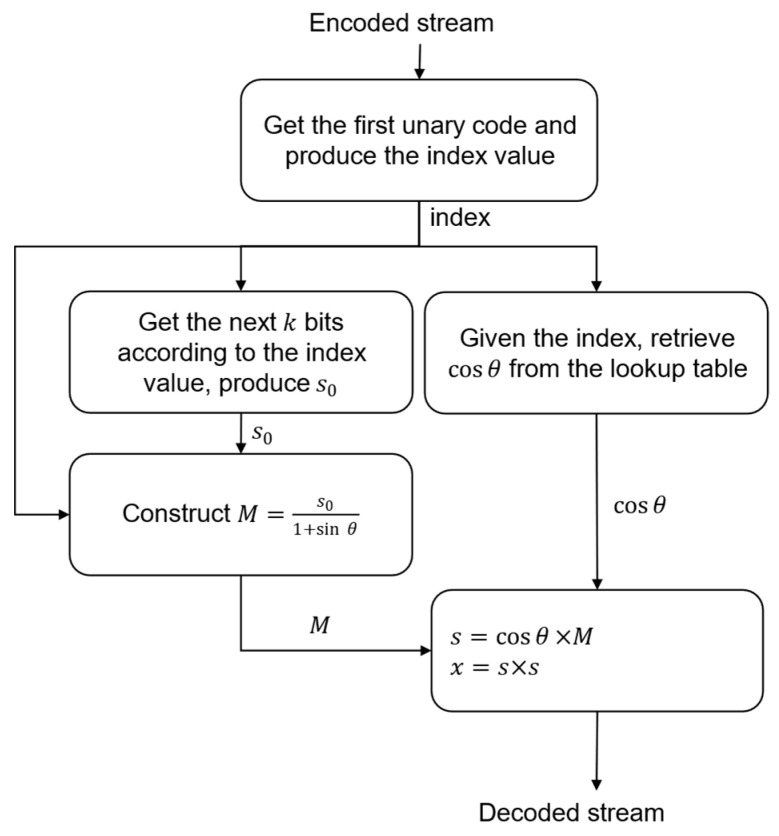
Steps representing the decoder side of the proposed near-lossless compression system.

Then, the square root value of x is reconstructed at the decoder by multiplying cos⁡θ with M. Squaring the resulting product gives the desired value of x. Figure 7 displays the different steps involved in the reconstruction of the original stream by the decoder.

Next, we disclose the pseudocodes for the near-lossless encoder and decoder in Algorithms 5 and 6, respectively.
**Algorithm 5.** Pseudocode for the compressor part of the proposed near-lossless compression.**Input:** x // hyperspectral data. **Outputs:** vecSeed, vecUnary. **Initialization:** n← the number of bits required to represent x. **For all** $x_{i}\;$in x **Do**        **1. Seed generation**        MSH ← the leftmost ⌈n/2⌉$\;\mathrm{bits}\;\mathrm{of}\;x_{i}$        $Q\leftarrow 2^{\left\lfloor n/2\right\rfloor }$        seed ← (MSH+Q)≫1        **2. Quadrature-based square rooting**        BC←seed        CD←x⁄BC        M←0.5⋅(BC+CD)        CM←BC−M        sin⁡θ←CM/M        **3. Preparing the compressed stream**        $index\leftarrow 10^{2}\cdot \sin\theta$        order ← the corresponding order of the seed value within the lookup table of the index (Table A1).        m ← the number of bits that correspond to index (Table 4).        varCode← the least significant m bits of order.        vecSeed.add(varCode) // add the encoded seed to vecSeed vector.        unary ← the corresponding unary code of the index value.        vecUnary.add(unary) // add unary code to vecUnary vector. **End Do**

**Algorithm 6.** Pseudocode for the decompressor part of the proposed near-lossless compression.**Inputs:** vecSeed, vecUnary **Output:** x // reconstructed hyperspectral data **Initialization:** index ← the index value obtained by interpreting the next unary code in vecUnary **For all** index **Do**        m← the number bits to be read from vecSeed based on index value (Table 4).        order ← get the next m bits from vecSeed.        seed← get the seed value given the index (Table A1).        cos⁡θ← given the index value (that corresponds to sin⁡θ value), retrieve the cosine value from the lookup table.        $\sin\theta \leftarrow index/10^{2}$        M←seed/(1+sin⁡θ)        s←cos⁡θ×M        x←s×s **End Do**


The sequential time complexity of the near-lossless algorithm is dominated by the seed generation and quadrature-based square rooting steps, as both involve basic arithmetic operations that contribute to a worst-case time complexity of O($n^{3}$). Unary encoding, which typically involves appending a sequence of 1s followed by a single 0, also plays a role in the algorithm’s time complexity. However, the use of a precomputed lookup table for the 24 indices and their corresponding unary codes would only require a constant time complexity, thus maintaining the overall complexity at O($n^{3}$). Overall, the entire algorithm can be considered to have a worst-case sequential time complexity of O($n^{3}$).

## 5. Experimental Results and Discussion

Our simulation results were obtained using the MATLAB computing environment R2020 installed on a MacBook Pro machine with a 2.4-GHz Apple M2 Max processor. This system has 32 GB of RAM and is running a macOS Ventura 13.3.1.

### 5.1. Dataset Description

**Figure 8:**
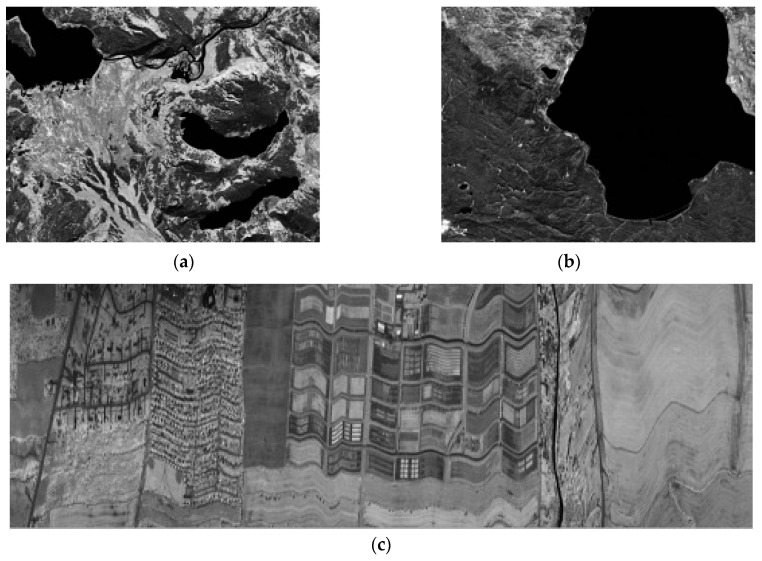
Three examples of hyperspectral scenes used in our experiments: (**a**) Yellowstone sc03, U (band 128); (**b**) Yellowstone sc10, U (band 128); (**c**) CASI t0477f06 U (band 64).

To ensure the reproducibility of the results and allow for comparison with other state-of-the-art works, the Corpus dataset is used for analysis [83]. Since this dataset represents a collection of both multispectral and hyperspectral images, only the latter type will be utilized in this work. Further, the Corpus dataset is heavily employed by the research community, including CCSDS, to evaluate the performance of compression algorithms [84]. The description of the 32 selected hyperspectral images is provided in Table 6. All files are stored in band sequential format (BSQ). The data types of these images are either u16be (unsigned 16-bit big-endian) or s16be (signed 16-bit big-endian) integers. These integers are represented using the two’s complement format [85]. In our study, the BSQ format is transformed into band interleaved by line (BIL) format to simulate the line-by-line acquisition of the underlying scene in real time. In Figure 8, we display three examples of hyperspectral scenes used in our experiments. The first two originate from the AVIRIS imager and the third belongs to CASI.

### 5.2. Results of Lossless Compression

The compression performance of the proposed lossless compressor is evaluated and compared to the compact data structure $k^{2}$-raster combined with directly addressable codes, as recently published in [40]. The k2-raster method is a technique for compactly representing raster data, such as images, using a hierarchical tree structure. The method begins by partitioning the matrix representing the raster data into square sub-quadrants of equal size. If the matrix cannot be perfectly partitioned into such sub-quadrants, it is enlarged to a size that can accommodate the partitioning. This enlarged matrix is then recursively partitioned until each quadrant contains cells with identical values or reaches a size of 1 × 1. This partitioning process forms a tree structure, represented as a bitmap. At each level of the tree, the maximum and minimum values of each quadrant are computed and compared with the parent values. The differences between the quadrant extrema and the parent extrema are stored in arrays. By storing differences instead of original values, the method facilitates compression. DAC is then used to compress $k^{2}$-raster data and provide access to variable-length codes. The time complexity to generate all $k^{2}$-rasters is equal to O($n^{3}$). This is besides the time complexity needed to query each cell in the tree structure, which is equal to O($\log_{k}n\cdot L$), where $k^{2}$ is the size of the sub-quadrant at each one of the L levels of the tree [62]. We selected this study because it shows thorough details of the compression performance and complete information regarding the selected images in the Corpus dataset. It would also allow for a one-to-one comparison of the compression results obtained for each HSI.

We first investigate the impact of using the XOR operation for decorrelating hyperspectral data characterized by the percentage of zero elements, also defined as sparsity. The increased sparsity is presented in Table 7 and Table 8 for a 16-bit and 8-bit data, respectively. As stated previously in Section 3, the zero elements must occupy more than 6.25% of the 16-bit image and 12.50% of the byte-partitioned image for any reduction to be obtained. These two tables show the impact of decorrelation and partitioning on the compression ratio for hyperspectral data produced by AVIRIS. The column labeled original sparsity provides sparsity values before data decorrelation. We observe that the two scenes Yellowstone (sc03, C) and Yellowstone (sc10, C) exhibit an interesting characteristic whereby their sparsity values after decorrelation remain nearly unchanged from their original values when using 16-bit representations, as displayed in Table 7. This is in contrast to their respective values when using 8-bit representations where there is, as expected, an increase in their sparsity after decorrelation (see Table 8). Our justification for these two occurrences is that the behavior of these two images, with respect to sparsity, may be influenced by their calibration status or inherent data characteristics. However, without detailed information from the dataset provider regarding the former factor, it is challenging to pinpoint the precise reasons involved. Moreover, the presence of noise or artifacts in the two images could also affect their sparsity levels, as they may introduce additional variations in pixel values. Since this behavior did not manifest itself again when the images are blocked into bytes, the sparsity increased significantly. This suggests that while the values across bands may exhibit similarities in their most significant bits (MSBs), the differences are significant enough such that when considering the entire 16 bits they may influence the resulting sparsity values after decorrelation.

The average bit rate used in finding the compression ratio is calculated using Equations (7) and (8). For instance, the percentage of zero elements of the Maine scene is 53.51% after decorrelation and blocking into eight bits. The calculated average bit rate, using Equation (8), is reduced from 8 to 4.7192, that is 0.5351×1+0.4649×9. As a result, the obtained compression ratio has reached a promising value of 8/4.7192=1.6952 by using preprocessing techniques for this specific image. As revealed in Table 7 and Table 8, the obtained compression ratio of the preprocessing step is higher with the adoption of byte-wise processing. The geometric mean of the compression ratio for all the selected hyperspectral images is 1.2 for the decorrelated 16-bit values and 1.5 for the decorrelated 8-bit values.

**Figure 9:**
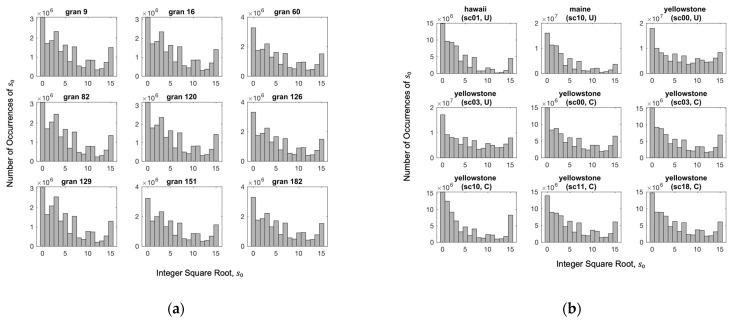
Histograms exhibiting the similarity in the distributions of the integer square roots, $s_{0}$, for multiple hyperspectral images generated by the two imagers: (**a**) AIRS and; (**b**) AVIRIS, respectively.

The decorrelated 8-bit values are passed next to the seed generator to produce an accurate estimate of the integer square root. We note that by partitioning the data into bytes, the generated error is completely avoided, and thus, no error compensation is required. Since square rooting reduces the number of bits into four, the value of $s_{0}\in \left[1,15\right]$. This is because all zero elements are removed in the preprocessing stage. The zero value is used to encode the integer square roots of the perfect square integers. When employing our seed generation approach, we observe that the distribution of the obtained integer square roots follows a certain pattern, especially for the same imager. Although the observed pattern is not geometrically distributed per se, its consistent shape allows for offline mapping between the Rice codes and the integer square roots. In this regard, Figure 9 displays the distribution of the integer square roots for nine images of the AIRS and AVIRIS instruments, respectively.

To maximize the benefit of Rice coding, we suggest following the descending order of the most frequent values of $s_{0}$ given in the histogram data. This order is obtained by finding the integer square root value that appears the most often, known as the mode, for the dataset within the same instrument. For analysis purposes, we consider both one-dimensional and two-dimensional XORing for the preprocessing stage. One-dimensional XORing involves computing the bitwise XOR operation between consecutive spectral values, that is across bands only. On the other hand, two-dimensional XORing is applied by calculating the bitwise XOR operation along both the spectral and spatial dimensions, that is, across bands and along the line of the scene. Table 9 shows the mapping of Rice codes to the integer square roots according to the data distributions of the AIRS and AVIRIS imagers. The goal is to directly access Rice codes of the obtained integer square root in a single clock cycle utilizing a small-sized lookup table. Direct addressing into lookup tables locates the desired entry in O(1) time [86]. Nonetheless, smaller lookup tables are preferable for area and power considerations. The required size of such a lookup table is 16 bytes only, including the 16 variations from 0 to 15, where each code has a maximum length of 8 bits. The value obtained from the lookup table is then concatenated to the calculated distance between x and $s_{0}^{2}$.

To find the reduction percentage, the total number of bits after compression is divided by the total number of bits of the original hyperspectral image. Then, the obtained value is subtracted from one. These results are compared to those yielded by the state-of-the-art $k^{2}$-raster method, as reported in [40]. This comparison is displayed in Table 10. We clearly observe that an improved reduction is realized when employing our proposed seed generation technique by the lossless compressor, especially with direct addressing of Rice codes. A compression ratio of up to 2.6 is achieved while maintaining a reasonable computational complexity. As observed from the table, the preprocessing of hyperspectral data using two-dimensional XORing is sometimes detrimental, although it shows the highest compression performance for a few images. This is confirmed by the geometric mean values of the results encoded using Rice codes. These values are 34.45% and 33.13% for the images decorrelated by employing 1D and 2D XORing, respectively. Similarly, the geometric mean values for the images encoded using mapped Rice are 36.89% and 35.72% for the images employing the same respective decorrelations. Furthermore, these values show that the best results are obtained when combining both 1D XORing and mapped Rice codes. Overall, the generated results by the four variations of our proposed method for lossless compression outperform those produced by the $k^{2}$-raster method [40]. Specifically, reduction enhancements ranging from 16.65% to 29.89% are achieved by all these variations when compared in terms of their geometric mean values with that obtained by the $k^{2}$-raster technique.

**Figure 10:**
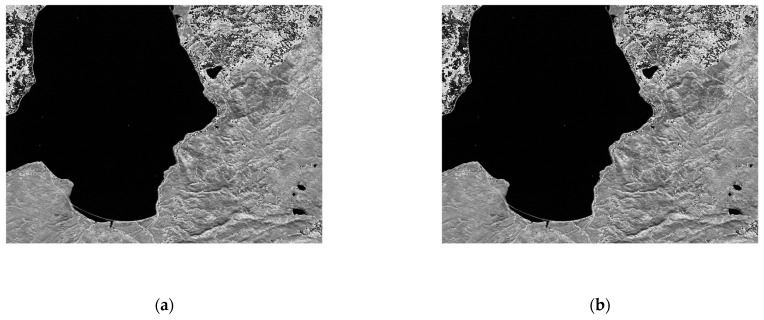
Visual comparison of applying our lossless compressor to AVIRIS Yellowstone (sc10, C) band 106: (**a**) original image; (**b**) reconstructed image.

Our proposed algorithm of lossless compression can be applied to power-of-two precision of the resolution values starting from 8 bits, then 16 bits, followed by 32 bits, and so on. All considered images in the dataset are stored using 16 bits with a 4-bit padding for those produced by a 12-bit instrument such as those by the AIRS instrument. Although the hyperspectral data are processed using a word length, we consider the actual bit rate of the imager when calculating the reduction percentage to maintain a fair comparison. For example, the total number of bits for the hyperspectral data produced by each AIRS scene is calculated as 1501×135×90×12 bits. The entropy results provided in the said table are calculated using the entropy model given by Equation (1). In Figure 10, we exhibit the original and the reconstructed images of the AVIRIS Yellowstone scene 10, calibrated (band 106) after applying our lossless compression and decompression algorithms. We note that the visual assessment of each image is extremely similar.

#### Comparison with Other Lossless Methods

We provide in Table 11 a comparison of our HSI lossless compression algorithm with other lossless methods in terms of bit rate (bpp). In addition to the four variations of our algorithm, we include in this table results from gzip (GNU Zip), bzip2, xz [62], and $k^{2}$-raster. The results show that our algorithm outperforms $k^{2}$-raster and gzip while its bit rate values are higher when compared to those obtained by the two methods: bzip2 and xz. The latter two techniques include transform-based algorithmic components which may make them amenable to yield lower bit rate values. In general, transform-based compression methods can offer superior compression ratios but can be computationally expensive, making them less suitable for real-time applications, where minimizing computational complexity and execution time is essential [87]. We have only included in the mentioned table the common scenes, from the Corpus dataset, having available bit rate values among all disclosed methods [62].

### 5.3. Results of Near-Lossless Compression

**Figure 11:**
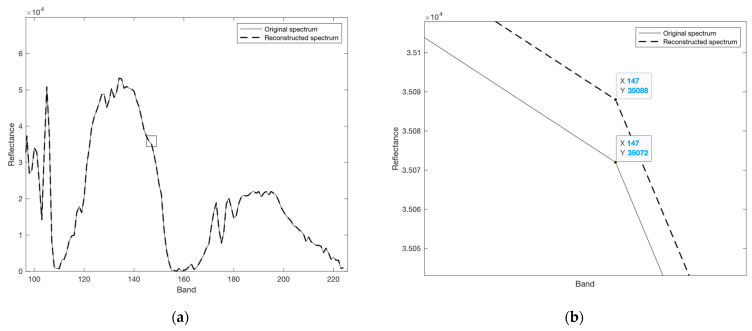
Original spectral data of the spatial location (100,100) of Yellowstone (sc18, C) image versus its reconstructed spectrum: (**a**) The spectral data showing selected bands from 100 to 224; (**b**) An enlarged plot showing the difference in magnitude between the original and the reconstructed value of band 147 at the same spatial location as indicated by the selection in (**a**) using a square shape.

For high entropy images that are hard to compress, we suggest employing our proposed quadrature-based square rooting method to realize acceptable compression ratios while maintaining highly accurate data for the decompressed stream. The compression performance based on this method is stabilized at a reduction of nearly 40% for real-world hyperspectral data. The near-lossless feature discussed in this work has a small to negligible impact on the accuracy of the decompressed data, as the nature of the produced error values reflects those generated by the square rooting method. This has a direct impact on preserving the shape of the original spectral signature of the image and is clearly seen in Figure 11 for the Yellowstone (sc18, C) image.

**Figure 12:**
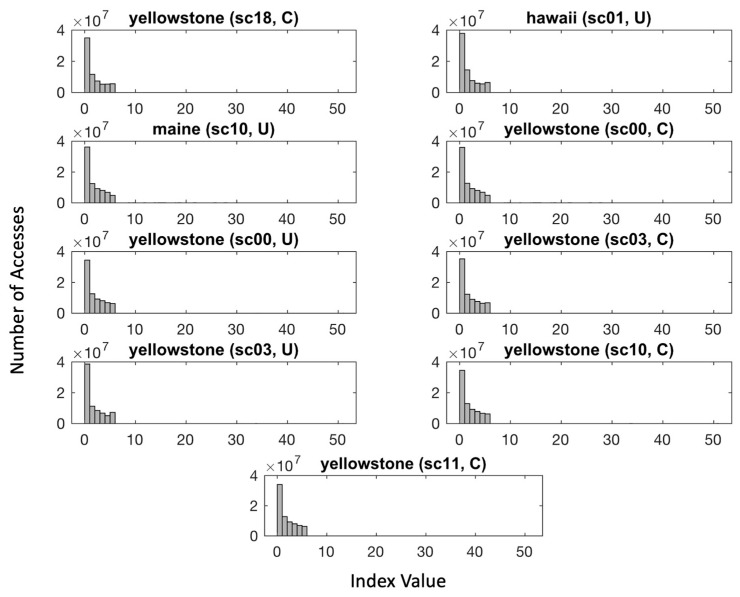
Histograms showing the number of accesses for each of the 24 indices, ranging from 0 to 50, of all 9 images produced by the AVIRIS imager.

The near-lossless compressor eventually produces the concatenation of the index value with the generated seed $s_{0}$. To further reduce the output stream, we replace the binary code of the index value with the corresponding unary code. As illustrated in Figure 12, the distributions of the indices are significantly skewed to the right for all AVIRIS test images. Statistically, the occurrence of the first few indices out of a total of 24 is more often than the occurrence of the remaining indices. Therefore, instead of concatenating five bits to represent the index, we can use the unary code instead and improve the average bit rate of the compressed stream.

Moreover, since the values of $s_{0}$ are unevenly distributed across the indices (see Table A1), we encode the 8-bit values of $s_{0}$ with its order within the index. This translates to a reduction in the number of bits required to represent $s_{0}$, given by n where 0≤n≤8. For instance, n is equal to zero for indices greater than or equal to 14 indicating a single occurrence of $s_{0}$. By applying the previous procedure, we disclose in Table 12 the data reduction percentage as well as the Maximum Relative Error (MRE) and the Maximum Absolute Error (MAE) for the selected dataset. All hyperspectral images that show a reduction percentage of less than 40%, when using lossless compression, are selected for processing by the near-lossless compressor. We calculated the maximum absolute error for the reconstructed values $\hat{x}$ using the following equation:

(12) $$MAE=max(\left|\hat{x}-x\right|).$$

Therefore, the maximum relative error is formulated as:

(13) $$MRE=max(\left|\hat{x}-x\right|/x).$$

**Figure 13:**
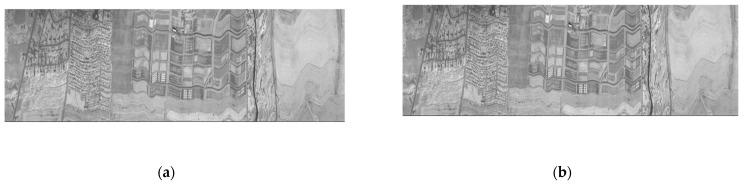
Near-lossless compression of CASI (t0477f06, U) band 70: (**a**) original image; (**b**) reconstructed image.

**Figure 14:**
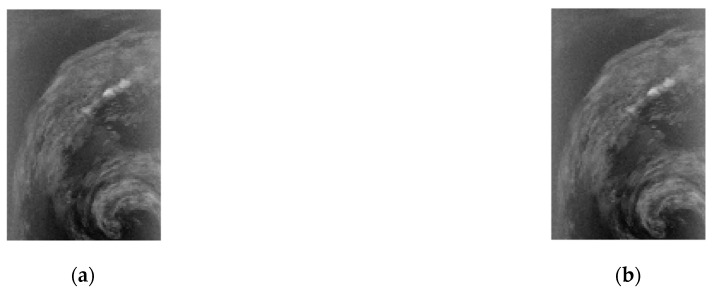
Near-lossless compression of AIRS (granule 16, U) band 208: (**a**) original image; (**b**) reconstructed image.

In both Figure 13 and Figure 14 below, we reveal the original and the reconstructed images of both the CASI uncalibrated image t0477f06 (band 70) and the AIRS uncalibrated granule 16 image (band 208) obtained after applying our near-lossless compressor, respectively. We can state that the visual assessment of each decompressed image with respect to its original one is highly similar.

#### Comparison with Other Near-Lossless Methods

When comparing to other works, vector quantization techniques were used previously in [30] to achieve near-lossless compression. This work shows high compression ratios of 20:1. The details of the generated error for the full dataset used in the study are not reported. However, the maximum absolute error reaches 127 when evaluating selected images in the AVIRIS dataset. Compared to the quadrature-based method, our results produce four times higher the accuracy. Furthermore, a recent study by Zheng et al. shows a significant reduction of at least 53% where the authors evaluated the compression performance by using three hyperspectral images of which only one is included in our dataset [25]. Nonetheless, the study relies on a recursive least-squares (RLS) filter with a loop quantizer, where the weight matrix is updated iteratively. RLS has a fast convergence rate, yet its computational complexity is in the order of $O(N^{2})$, which implies major limitations to its applications [88,89]. This is particularly true for power-critical systems, such as onboard computers required to process large volumes of hyperspectral data. Accordingly, our proposed quadrature-based method may be more feasible for onboard compression as it is highly parallelizable and needs only a single-pass for data processing while exhibiting minimum memory requirements.

## 6. Conclusions

Two new methods for lossless and near-lossless compression of remotely sensed hyperspectral images are reported in this article. We achieved lossless compression by employing our recent method of seed generation based on bit-manipulation techniques. Due to its acceptable complexity, the method could prove to be very efficient for lossless compression of hyperspectral images, where power and computational resources could be confined onboard satellites. Four variations of this technique are considered yielding a compression ratio of up to 2.6 while outperforming a state-of-the-art method, known as $k^{2}$-raster, by as much as 29.89% in terms of data reduction obtained by using HSIs from the Corpus dataset.

For those instances when hyperspectral images are hard to losslessly compress due to their reduced correlation, we suggest employing our near-lossless compression technique, which relies on our previously described quadrature-based square rooting method. Its obtained compression performance is stabilized at nearly 40% (from 38.9011% to 39.7314%) reduction of the original data within a maximum relative error of 0.33 and a maximum absolute error of only 30.

For hyperspectral data compression onboard satellites, we recommend the utilization of an adaptive lossless and near-lossless compression scheme, whereby a selection criterion is adopted based on a threshold value set by the user to indicate the acceptable level of compression performance. As part of our future work, we intend to implement both compression techniques on hardware accelerators, such as FPGA boards, commonly used for image processing.

## Figures and Tables

**Table 1:** Overview of recent studies related to HSI compression.

Reference	Method	Category	Type	Year
[40]	$k^{2}$-raster	Compact Data Structure	Lossless	2020
[45]	HCCNet	Deep Learning	Lossy	2023
[44]	Autoencoders	Deep Learning	Lossy	2021
[43]	SVR	Machine Learning	Lossy	2020
[42]	RNN	Deep Learning	Lossless	2019
[41]	CNN	Deep Learning	Lossy	2019
[48]	HyperLCA	Transform-Based	Lossy	2022
[22]	HW-HyperLCA	Transform-Based	Lossy	2019
[49]	3D-WBTC	Transform-Based	Lossy	2019
[51]	Spectral Graph Transform	Transform-Based	Lossless	2019
[50]	3D-DCT	Transform-Based	Lossy	2018
[52]	Tensor-Robust CUR	Tensor-Based	Lossy	2023
[54]	Tucker Decomposition	Tensor-Based	Lossy	2021
[59]	Optimized CS	Compressed Sensing	Lossy	2020
[58]	CACS	Compressed Sensing	Lossy	2019
[57]	SHSIR	Compressed Sensing	Lossy	2019
[56]	BlockSparse Dictionary	Compressed Sensing	Lossy	2018
[60]	B-CRLS	Recursive Least-Squares	Lossless	2018
[61]	SuperRLS	Recursive Least-Squares	Lossless	2018
[61]	BSuperRLS	Recursive Least-Squares	Lossless	2018

**Table 2:** Number of bits required to represent the fractional part relative to the integer square root value.

$s_{0}$	Fractional Bits
$2^{0}$	1
$2^{1}$	2
$2^{1}<s_{i}\leq 2^{2}$	3
$2^{2}<s_{i}\leq 2^{3}$	4
$2^{3}<s_{i}\leq 2^{4}$	5
$2^{4}<s_{i}\leq 2^{5}$	6
$2^{5}<s_{i}\leq 2^{6}$	7
$2^{6}<s_{i}\leq 2^{7}$	8
$2^{7}<s_{i}\leq 2^{8}$	9

**Table 3:** Codewords of the lossless square root-based encoder.

x	$s_{0}$	Integer Bits	Fractional Bits
1	1	0000	0 (unary)
2	1	0001	0
3	1	0001	1
4	2	0000	10 (unary)
5	2	0010	00
6	2	0010	01
7	2	0010	10
8	2	0010	11
9	3	0011	00
...	...	...	...
16	4	0000	110 (unary)
17	4	0100	000
18	4	0100	001
19	4	0100	010
20	4	0100	011
21	4	0100	100
22	4	0100	101
23	4	0100	110
24	4	0100	111
25	5	0101	0000
...	...	...	...

**Table 4:** Number of bits required to represent the variations of $s_{0}$ for each of the 24 index values.

Index	$\mathrm{Variations}\;\mathrm{of}\;s_{0}$	Number of Bits
0	157	8
1	111	7
2	83	7
3	66	7
4	52	6
5	31	5
6	7	3
7	5	3
8	4	2
9	5	3
10	3	2
11	4	2
12	2	1
13	2	1
14	1	0
15	1	0
17	1	0
18	1	0
20	1	0
21	1	0
25	1	0
27	1	0
33	1	0
50	1	0

**Table 5:** Sequence of ten 16-bit integers x with both their $s_{0}$ values and corresponding indices.

x	$s_{0}$	Index
29	5	7
12,317	112	0
22,556	152	1
31,003	185	4
5403	74	0
1818	44	3
21,017	146	0
61,974	249	0
15,125	123	0
10,260	104	2

**Table 6:** Description of the hyperspectral images within the Corpus dataset, selected for the evaluation of compression performance.

Imager	Scene	Data Type	Dimensions	C\U *	Bit Rate
AIRS	gran9	u16	1501 × 135 × 90	U	12
	gran16		1501 × 135 × 90		
	gran60		1501 × 135 × 90		
	gran82		1501 × 135 × 90		
	gran120		1501 × 135 × 90		
	gran126		1501 × 135 × 90		
	gran129		1501 × 135 × 90		
	gran151		1501 × 135 × 90		
	gran182		1501 × 135 × 90		
AVIRIS	Hawaii	u16	224 × 512 × 614	U	16
	Maine		224 × 512 × 680		
	Yellowstone (sc00)		224 × 512 × 680		
	Yellowstone (sc03)		224 × 512 × 680		
AVIRIS	Yellowstone (sc00)	s16	224 × 512 × 677	C	16
	Yellowstone (sc03)		224 × 512 × 677		
	Yellowstone (sc10)		224 × 512 × 677		
	Yellowstone (sc11)		224 × 512 × 677		
	Yellowstone (sc18)		224 × 512 × 677		
CRISM	sc182	u16	545 × 450 × 320	U	12
	sc214		74 × 2700 × 64		
CASI	t0477f06	u16	72 × 1225 × 406	U	12
	t0180f07		72 × 2852 × 405		
Hyperion	Cuprite	u16	242 × 1024 × 256	U	12
	ErtaAle		242 × 3187 × 256		
	LakeMonona		242 × 3176 × 256		
	MtStHelens		242 × 3242 × 256		
M3	globalA	u16	86 × 512 × 320	U	12
	globalB		86 × 512 × 320		
	targetA		260 × 512 × 640		
	targetB		260 × 512 × 640		
	targetC		260 × 512 × 640		
SFSI	Mantar	u16	240 × 140 × 496	U	12

* Calibrated (C)/Uncalibrated (U).

**Table 7:** The impact of data decorrelation on the sparsity of the hyperspectral data, represented by the percentage of zero elements, when using 16 bits for the AVIRIS dataset.

Scene	Original Sparsity	Sparsity after Decorrelation	Average Bit Rate	CR
Hawaii (U)	01.45%	25.84%	12.87	1.2
Maine (U)	0%	25.90%	12.86	1.2
Yellowstone (sc00, C)	0%	30.80%	12.07	1.3
Yellowstone (sc00, U)	01.19%	19.86%	13.82	1.2
Yellowstone (sc03, C)	0%	00.19%	16.97	0.9
Yellowstone (sc03, U)	02.65%	24.69%	13.05	1.2
Yellowstone (sc10, C)	0%	00.18%	16.97	0.9
Yellowstone (sc11, C)	07.68%	35.58%	11.31	1.4
Yellowstone (sc18, C)	02.03%	27.12%	12.66	1.3

**Table 8:** The impact of data decorrelation on the sparsity of the hyperspectral data, represented by the percentage of zero elements, when using 8 bits for the AVIRIS dataset.

Scene	Original Sparsity	Sparsity after Decorrelation	Average Bit Rate	CR
Hawaii (U)	04.74%	43.05%	5.56	1.4
Maine (U)	11.77%	53.51%	4.72	1.7
Yellowstone (sc00, C)	14.28%	54.23%	4.66	1.7
Yellowstone (sc00, U)	04.43%	43.82%	5.49	1.5
Yellowstone (sc03, C)	00.96%	27.52%	6.80	1.2
Yellowstone (sc03, U)	06.53%	44.91%	5.41	1.5
Yellowstone (sc10, C)	01.33%	28.85%	6.69	1.2
Yellowstone (sc11, C)	14.12%	52.40%	4.81	1.7
Yellowstone (sc18, C)	05.80%	47.85%	5.17	1.5

**Table 9:** Suggested order of mapping the integer square roots to Rice codes using 1D and 2D XORing in the preprocessing stage.

Rice Code	AIRS-1D	AIRS-2D	AVIRIS-1D	AVIRIS-2D
0.0	0	0	0	0
0.1	3	3	1	1
10.0	2	2	2	2
10.1	1	1	15	3
110.0	5	5	3	5
110.1	7	7	7	15
1110.0	15	15	5	7
1110.1	4	4	14	4
11110.0	10	11	10	10
11110.1	14	10	4	11
111110.0	6	6	6	6
111110.1	11	14	11	14
1111110.0	9	8	9	8
1111110.1	13	9	13	9
11111110.0	8	13	8	13
11111110.1	12	12	12	12

**Table 10:** Data reduction percentage of four variations of our proposed method for lossless compression and their comparison with results obtained using $k^{2}$-raster [40]. Higher reduction values are displayed in boldface. The geometric mean values and the achieved enhancements in data reduction of the four variations are displayed in the last two rows.

Imager	Scene	Entropy (Bits)	$k^{2}$-Raster	Proposed (1D XOR, Rice)	Proposed (2D XOR, Rice)	Proposed (1D XOR, Mapped Rice)	Proposed (2D XOR, Mapped Rice)
AIRS	gran9	11.2	21%	22%	23%	25%	**26%**
	gran16	11.1	24%	24%	24%	**26%**	**26%**
	gran60	11.5	19%	18%	20%	20%	**22%**
	gran82	11.0	-	29%	27%	**32%**	30%
	gran120	11.2	-	25%	25%	**27%**	**27%**
	gran126	11.5	20%	20%	21%	22%	**24%**
	gran129	11.1	28%	31%	29%	**34%**	31%
	gran151	11.6	21%	23%	23%	**26%**	25%
	gran182	11.6	19%	19%	20%	**22%**	**22%**
AVIRIS	Hawaii	8.6	-	58%	57%	**59%**	57%
	Maine	9.1	-	**58%**	57%	**58%**	57%
	Yellowstone (sc00, U)	12.6	**25%**	19%	22%	22%	**25%**
	Yellowstone (sc03, U)	12.3	**27%**	22%	25%	24%	**27%**
AVIRIS	Yellowstone (sc00, C)	10.3	40%	39%	43%	41%	**44%**
	Yellowstone (sc03, C)	9.9	41%	40%	44%	43%	**46%**
	Yellowstone (sc10)	8.6	52%	53%	52%	**55%**	**55%**
	Yellowstone (sc11)	9.8	45%	46%	48%	47%	**49%**
	Yellowstone (sc18)	10.2	39%	39%	44%	41%	**46%**
CRISM	sc182	11.2	16%	35%	27%	**37%**	29%
	sc214	9.9	-	60%	52%	**61%**	53%
CASI	t0477f06	10.4	-	24%	23%	**27%**	25%
	t0180f07	10.7	-	15%	17%	18%	**19%**
Hyperion	Cuprite	9.4	-	44%	37%	**46%**	40%
	ErtaAle	9.5	35%	43%	36%	**45%**	38%
	LakeMonona	9.9	35%	43%	36%	**45%**	38%
	MtStHelens	9.3	34%	40%	33%	**42%**	36%
M3	globalA	9.4	-	44%	37%	**46%**	43%
	globalB	9.3	-	45%	38%	**47%**	45%
	targetA	8.7	-	55%	48%	**56%**	51%
	targetB	9.7	-	52%	45%	**53%**	48%
	targetC	8.8	-	61%	54%	**62%**	56%
SFSI	mantar	7.2	-	47%	40%	50%	45%
Geometric Mean			28.40%	34.45%	33.13%	**36.89%**	35.72%
Reduction Enhancement			NA	21.30%	16.65%	**29.89%**	25.77%

**Table 11:** Bit rate values of four variations of our proposed method for HSI lossless compression and their comparison with results obtained using $k^{2}$-raster [40], gzip, bzip2, and xz [62] for a subset of the Corpus dataset.

Imager	Scene	Proposed (1D XOR, Rice)	Proposed (2D XOR, Rice)	Proposed (1D XOR, Mapped Rice)	Proposed (2D XOR, Mapped Rice)	$k^{2}$-Raster (DACs)	gzip	bzip2	xz
AIRS	gran9	9.37	9.23	9.03	8.92	9.49	10.16	7.42	7.90
	gran16	9.16	9.13	8.82	8.83	9.12	9.82	7.15	7.66
	gran60	9.89	9.63	9.56	9.33	9.72	10.53	7.71	8.23
	gran126	9.65	9.47	9.33	9.16	9.61	10.33	7.64	8.10
	gran129	8.25	8.57	7.94	8.26	8.65	9.50	6.68	7.22
	gran151	9.23	9.24	8.91	8.93	9.53	10.31	7.43	7.97
	gran182	9.72	9.60	9.39	9.29	9.68	10.64	7.79	8.33
AVIRIS	Yellowstone (sc00, U)	12.94	12.41	12.51	12.07	11.92	12.39	9.99	10.61
	Yellowstone (sc03, U)	12.46	11.95	12.11	11.63	11.74	11.98	9.54	10.23
AVIRIS	Yellowstone (sc00, C)	9.83	9.18	9.47	8.90	9.61	10.12	7.51	8.04
	Yellowstone (sc03, C)	9.53	8.89	9.15	8.57	9.42	9.59	7.10	7.62
	Yellowstone (sc10)	7.55	7.62	7.15	7.18	7.62	7.41	5.30	5.73
	Yellowstone (sc11)	8.72	8.38	8.45	8.11	8.81	9.04	6.65	7.07
	Yellowstone (sc18)	9.80	8.91	9.48	8.65	9.78	10.00	7.45	7.95
CRISM	sc182	7.83	8.81	7.57	8.53	10.11	10.90	8.53	7.90
Hyperion	ErtaAle	6.82	7.67	6.57	7.40	7.76	8.69	6.41	6.73
	LakeMonona	6.84	7.73	6.56	7.43	7.82	8.69	6.46	6.74
	MtStHelens	7.18	7.95	6.93	7.69	7.91	8.26	6.28	6.48

**Table 12:** Compression performance of the quadrature-based near-lossless HSI compressor, characterized by the data reduction percentage, MRE, and MAE values.

Imager	Scene	Data Reduction (%)	MRE	MAE
AIRS	gran9	39.4242	0.0667	30
	gran16	39.7075	0.0038	30
	gran60	39.5578	0.3333	30
	gran82	39.6562	0.0038	30
	gran120	39.5115	0.0667	30
	gran126	39.5593	0.0667	30
	gran129	39.6236	0.0038	30
	gran151	39.5363	0.3333	30
	gran182	39.5240	0.0667	30
AVIRIS	Yellowstone (sc00, U)	39.7314	0.0667	30
	Yellowstone (sc03, U)	39.7106	0.0667	30
CRISM	sc182	39.4889	0.3333	30
CASI	t0477f06	39.6337	0.3333	30
	t0180f07	38.9011	0.3333	30

## Data Availability

The dataset used in this research is publicly available at: https://cwe.ccsds.org/sls/docs/SLS-DC/123.0-B-Info/TestData/ (accessed on 4 July 2023).

## Appendix A

This appendix contains two tables: one providing the mapping of $s_{0}$ values to the 24 indices of cos⁡θ, followed by a second one giving the MATLAB code to generate such results.

**Table A1:** Nonuniform mapping of $s_{0}$ values to the 24 indices of cos⁡θ in the lookup table.

Index	$s_{0}$
0	0, 1, 2, 3, 4, 5, 6, 7, 8, 9, 10, 11, 12, 13, 14, 15, 16, 17, 18, 19, 20, 21, 22, 24, 25, 26, 27, 28, 29, 30, 31, 32, 33, 34, 35, 36, 37, 38, 39, 40, 41, 51, 52, 53, 54, 55, 56, 57, 58, 59, 60, 61, 62, 63, 64, 65, 66, 67, 68, 69, 70, 71, 72, 73, 74, 75, 76, 77, 78, 79, 107, 108, 109, 110, 111, 112, 113, 114, 115, 116, 117, 118, 119, 120, 121, 122, 123, 124, 125, 126, 127, 128, 129, 130, 131, 132, 133, 134, 135, 136, 137, 138, 139, 140, 141, 142, 143, 144, 145, 146, 147, 148, 149, 150, 151, 152, 153, 154, 155, 218, 219, 220, 221, 222, 223, 224, 225, 226, 227, 228, 229, 230, 231, 232, 233, 234, 235, 236, 237, 238, 239, 240, 241, 242, 243, 244, 245, 246, 247, 248, 249, 250, 251, 252, 253, 254, 255
1	5, 6, 7, 8, 9, 10, 11, 12, 13, 14, 15, 16, 17, 18, 19, 20, 21, 22, 23, 24, 25, 26, 27, 28, 29, 30, 31, 32, 33, 34, 35, 36, 37, 38, 39, 40, 41, 42, 43, 50, 51, 52, 53, 54, 55, 56, 57, 58, 59, 60, 61, 62, 63, 64, 65, 66, 67, 68, 69, 75, 76, 77, 78, 79, 80, 81, 82, 83, 84, 103, 104, 105, 106, 107, 108, 109, 110, 111, 112, 149, 150, 151, 152, 153, 154, 155, 156, 157, 158, 159, 160, 161, 162, 163, 164, 209, 210, 211, 212, 213, 214, 215, 216, 217, 218, 219, 220, 221, 222, 223, 224
2	5, 6, 7, 8, 9, 10, 11, 12, 13, 14, 15, 16, 17, 18, 19, 20, 21, 22, 23, 24, 25, 26, 27, 28, 29, 30, 31, 32, 33, 34, 35, 40, 41, 42, 43, 44, 45, 48, 49, 50, 51, 52, 53, 80, 81, 82, 83, 84, 85, 86, 87, 88, 100, 101, 102, 103, 104, 105, 106, 160, 161, 162, 163, 164, 165, 166, 167, 168, 169, 170, 171, 172, 203, 204, 205, 206, 207, 208, 209, 210, 211, 212, 213
3	4, 5, 7, 8, 9, 10, 11, 12, 13, 14, 15, 16, 17, 18, 22, 23, 24, 25, 29, 30, 31, 43, 44, 45, 46, 47, 48, 49, 50, 51, 85, 86, 87, 88, 89, 90, 91, 97, 98, 99, 100, 101, 102, 103, 169, 170, 171, 172, 173, 174, 175, 176, 177, 178, 179, 180, 197, 198, 199, 200, 201, 202, 203, 204, 205, 206
4	6, 7, 8, 9, 10, 12, 13, 14, 15, 16, 17, 18, 23, 24, 25, 45, 46, 47, 48, 49, 50, 89, 90, 91, 92, 93, 94, 95, 96, 97, 98, 99, 100, 178, 179, 180, 181, 182, 183, 184, 185, 186, 187, 188, 193, 194, 195, 196, 197, 198, 199, 200
5	3, 4, 5, 6, 7, 8, 9, 10, 12, 13, 14, 15, 16, 24, 47, 48, 93, 94, 95, 96, 97, 186, 187, 188, 189, 190, 191, 192, 193, 194, 195
6	6, 7, 8, 9, 13, 14, 15
7	5, 6, 7, 8, 9
8	4, 6, 7, 8
9	3, 5, 6, 7, 8
10	5, 7, 8
11	2, 4, 6, 7
12	6, 7
13	4, 6
14	3
15	4
17	4
18	3
20	2
21	3
25	3
27	2
33	1
50	1

**Table A2:** MATLAB code for generating the values displayed in Table A1.

function S0=LUT X=0:2^16−1; % SEED GENERATION OF s0 BASED ON BIT MANIPULATION nBits=max(1,floor(log2(X))+1); nShifts=floor(0.5 ∗ nBits); MSH=bitshift(X,−nShifts); s0=floor(0.5 ∗ (MSH+2.^nShifts)); % QUADRATURE−BASED SQUARE ROOTING BC=s0; CD=X./BC; CD(isnan(CD))=0; % HANDLING DIVISION BY ZERO M=0.5.∗ (BC+CD); CM=abs(BC−M); idx=floor((CM./M).∗ 10^2)+1; % INDEXING STARTS AT (1) idx(isnan(idx))=1; for i=1:51 % STEP SIZE OF 0.01 I=find(idx==i); S0{i}=unique(s0(I)); end end
